# Bio-Layer Interferometry Analysis of the Target Binding Activity of CRISPR-Cas Effector Complexes

**DOI:** 10.3389/fmolb.2020.00098

**Published:** 2020-05-27

**Authors:** Hanna Müller-Esparza, Manuel Osorio-Valeriano, Niklas Steube, Martin Thanbichler, Lennart Randau

**Affiliations:** ^1^Department of Biology, University of Marburg, Marburg, Germany; ^2^Max Planck Fellow Group “Bacterial Cell Biology”, Max Planck Institute for Terrestrial Microbiology, Marburg, Germany; ^3^Center for Synthetic Microbiology (SYNMIKRO), Marburg, Germany

**Keywords:** CRISPR-Cas, cascade, bio-layer interferometry, affinity, DNA-binding

## Abstract

CRISPR-Cas systems employ ribonucleoprotein complexes to identify nucleic acid targets with complementarity to bound CRISPR RNAs. Analyses of the high diversification of these effector complexes suggest that they can exhibit a wide spectrum of target requirements and binding affinities. Therefore, streamlined analysis techniques to study the interactions between nucleic acids and proteins are necessary to facilitate the characterization and comparison of CRISPR-Cas effector activities. Bio-layer Interferometry (BLI) is a technique that measures the interference pattern of white light that is reflected from a layer of biomolecules immobilized on the surface of a sensor tip (bio-layers) in real time and in solution. As streptavidin-coated sensors and biotinylated oligonucleotides are commercially available, this method enables straightforward measurements of the interaction of CRISPR-Cas complexes with different targets in a qualitative and quantitative fashion. Here, we present a general method to carry out binding assays with the Type I-Fv complex from *Shewanella putrefaciens* and the Type I-F complex from *Shewanella baltica* as model effectors. We report target specificities, dissociation constants and interactions with the Anti-CRISPR protein AcrF7 to highlight possible applications of this technique.

## Introduction

CRISPR-Cas systems are adaptive immune systems found in Archaea and Bacteria. They are widespread and diverse, with 6 types (I–VI) and 33 different subtypes described so far (Makarova et al., 2020). They are able to establish immunity against invading genetic material through ribonucleoprotein (RNP) complexes, formed by a CRISPR RNA (crRNA) and CRISPR-associated (Cas) proteins, in a process termed CRISPR interference (Barrangou et al., 2007). CRISPR-Cas types are classified by unique Cas proteins, which are often part of distinct RNP complexes and convey specific features and mechanistic variations.

In line with the described diversity of CRISPR-Cas complexes, different systems can exhibit distinct target requirements. Type I, II, and V complexes scan for DNA targets and first recognize a 2–5 bp short motif next to the region with complementarity to the crRNA (protospacer), termed protospacer adjacent motif (PAM) (Anders et al., 2014; Hayes et al., 2016; Yang et al., 2016). This motif is not present in the DNA sequence that codes for the crRNA, the CRISPR array, acting as a safety mechanism to avoid self-targeting. RNA-interacting Type III and VI complexes employ an exclusion mechanism and interfere with complementary sequences flanked by any motif but the one present in the CRISPR array (Marraffini and Sontheimer, 2010; Abudayyeh et al., 2016). A single system might be able to interact with several motifs with varying efficiencies and interference activities (Hayes et al., 2016).

The reported affinities of effector complexes for correct targets are also diverse. Published equilibrium dissociation constants (K_D_) include: Cas9 (Type II): 0.5 nM (Sternberg et al., 2014), Type V-A: 54 fM (Strohkendl et al., 2018), Type III-A: 0.1 nM (Mogila et al., 2019), Type I-F: 1 nM (Rollins et al., 2015), and Type I-E: 13 nM or 20.7 nM (Westra et al., 2012; Beloglazova et al., 2015). In addition, CRISPR-Cas complexes show varying tolerance toward mismatches between the target and the crRNA. It has been established as a general principle that the further away the mismatch is located from the PAM the more likely it is to be allowed (Zheng et al., 2017; Chen et al., 2018; Xue and Sashital, 2019). Despite this general principle, different complex types show variations in their interference mechanisms due to the involvement of a variety of diverse Cas proteins.

Due to this high variability, effector complex-target interactions should be studied on a case-by-case basis. Several approaches can be used to analyze how effector complexes interact with their targets. The most common *in vitro* method is the Electrophoretic Mobility Shift Assay (EMSA), but novel technologies involving label-free analytes and real-time measurements of interactions in solution can provide technical advantages. One of these new developments is Bio-layer Interferometry (BLI), which measures the interference pattern obtained on combination of white light reflected from a bio-layer and an internal reference surface. This technique is based on the use of a biosensor with a coated tip (bio-layer), on which a bait molecule can be immobilized. Subsequently, the interaction of a molecule of interest (analyte) with the bait can be monitored in real time by recording changes in light interference, which correlate directly with variations in the thickness of the biolayer resulting from the association of the analyte. The variety of commercially available biosensors allows studies of a wide range of interactions, for example between CRISPR-Cas complexes and their DNA or RNA substrates (Richardson et al., 2016; Shin et al., 2017). BLI can provide information on the affinity and stability of interactions and determine the rate constants of the binding reactions (Abdiche et al., 2008). Moreover, it allows for the evaluation of the interplay between CRISPR-Cas complexes and other proteins, such as anti-CRISPRs (Acrs) or nucleases, as it can measure sequential binding events that lead to the formation of super-complexes.

Here, we report the use of BLI to study the target interactions of two model Type I complexes. First, the target binding behavior of the Type I-Fv effector complex (Cascade) of *Shewanella putrefaciens* CN-32 (Dwarakanath et al., 2015) is analyzed. This complex contains three Cas proteins in addition to the crRNA, Cas6f, Cas7fv, and Cas5fv, with a 1:6:1 stoichiometry yet lacks a large subunit. The large subunit has been reported to be responsible for PAM recognition and dsDNA separation in I-E and I-F systems (Hayes et al., 2016; Chowdhury et al., 2017; Xiao et al., 2017). Cas5fv and Cas7fv have no sequence similarity to other described Cas proteins. Previous work has shown that these diversified proteins fulfill the tasks of the missing large subunit (Pausch et al., 2017), but it is unknown whether this different composition affects the way in which the complex binds to its targets and the affinity of the interaction.

Second, we study a Type I-F Cascade from *Shewanella baltica* OS195 to compare how the differences in complex architecture affect the interaction with targets. This system has the same CRISPR array structure as the Type I-Fv system, with 32 nucleotide spacers and identical repeat sequences, but it retains the protein architecture of canonical Type I-F systems and includes a large subunit (Supplementary Figure S1; Chowdhury et al., 2017; Couvin et al., 2018).

**FIGURE 1:**
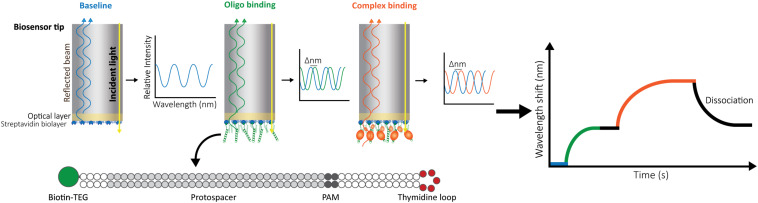
Schematic representation of Bio-layer Interferometry (BLI). BLI measures the shifts in reflected white light upon changes in the thickness of the bio-layer. The measured shift depends both on the size and the affinity of the interactors. Bio-layers are coated with molecules (e.g., streptavidin) that allow the immobilization of one of the interactors. For this study, 5′-biotin-tagged oligonucleotides were used. The long dsDNA oligonucleotides have complementary arms separated by a 5-thymidine loop to allow for dsDNA hybridization. In addition, they contain a sequence complementary to the crRNA carried by the effector complex (protospacer) and a protospacer-adjacent motif (PAM). After baselining the light signal on buffer (blue), the oligonucleotide is added, binding to the streptavidin and generating a shift (green) that is also baselined by incubating the bio-layer back in buffer (black straight line). After signal stabilization, the label-free ribonucleoprotein complex is added, and the generated shift is recorded until equilibrium is reached (orange). Dissociation of the complex is then measured by transferring the bio-layer back into buffer (black curve).

In our setup, we use a BLItz system (FortéBio) with single-use High Precision Streptavidin (SAX) Biosensors (Dip and Read^TM^, FortéBio). We measure the interaction as follows: after measuring the background signal from the buffer, we immobilize one interactor, for instance a biotinylated double-stranded oligonucleotide, on the streptavidin-coated biosensor and set a baseline. Then, we monitor the change in the wavelength shift induced by association of the second interactor (a CRISPR-Cas effector) until the equilibrium is reached. Finally, the biosensor is incubated in a large volume of buffer to follow the dissociation of Cas proteins. The wavelength change observed reflects the variation in the thickness of the bio-layer due to the binding or detachment of molecules (Figure 1).

This method allows for (i) obtaining equilibrium dissociation constants (K_D_) for the interactions between Cascades and dsDNA, (ii) the qualitative analysis of target requirements, including PAM and sequence complementarity, and (iii) an investigation of the effect of a predicted Acr inhibitor protein on Cascade binding. These applications of BLI to study model Type I-F and I-Fv complexes highlight the usefulness of this technique for dissecting the target preferences and targeting mechanisms of CRISPR-Cas systems.

## Materials and Methods

### Strains and Plasmids

Strain BL21(DE3)pLysS (Agilent Technologies) was used for the overproduction of Cascade complexes and Anti-CRISPR proteins. Strain BL21-AI (Invitrogen) was used for Efficiency of Transformation Assays (EOT). The plasmids used in this study are indicated in Supplementary Table S1. All strains were grown in LB medium supplemented with the corresponding antibiotics.

For purification of the I-Fv Cascade, previously described plasmids were used (Gleditzsch et al., 2016). The Cas operon from *Shewanella putrefaciens* (*cas2-3, cas7fv, cas5fv, cas6f*) was further subcloned under a rhamnose inducible promoter in a pUC backbone (iGEM part BBa_K914003) for *in vivo* assays.

For expression of the *Shewanella baltica* OS195 system in *E. coli*, the I-F *cas* genes were amplified via PCR from genomic DNA. *cas2-3* and Cascade genes were generated as independent cassettes. The *cas2-3* gene was integrated into the second multiple cloning site of a pCDFDuet-1 vector carrying a minimal CRISPR array with a single spacer against the ampicillin resistance cassette of pETDuet-1. Cascade genes were assembled as an operon in a pACYCDuet-1 vector via Gibson assembly. The HD domain of Cas2-3 was identified by sequence comparison with the Cas2-3 of *S. putrefaciens* and alanine replacement of this domain was carried out through site-directed mutagenesis. For protein purification, a sequence encoding a His-tag was added to the 5′ end of *cas7f* by inverse fusion PCR cloning. The activity of the tagged complex was confirmed by Efficiency of Transformation assays.

The gene for an AcrF7 variant carrying an N-terminal His-tag was subcloned from a pHERD30T plasmid (provided by Dr. Alan Davidson, Pawluk et al., 2016) into the first multiple cloning site of pRSFDuet-1.

### Efficiency of Transformation Assays

To test the activity of the *S. baltica* and *S. putrefaciens* Cascade complexes, *E. coli* BL21-AI was transformed with plasmids encoding Cascade components and wild-type Cas2-3 or an inactive Cas2-3 HD domain variant (C466G, A467C, A470C mutations in *cas2-3*). The resulting strain was then transformed with a plasmid carrying a minimal CRISPR with a spacer targeting the ampicillin resistance cassette of pETDuet-1 (spacer sequence: 5′-AGTCACAGAAAAGCATCTTACGGATGGCATGA-3′, Pausch et al., 2017). A pRSFDuet-1 derivative encoding AcrF7 was added in some experiments as indicated. The plasmid carrying the *acr* gene used for sub-cloning was kindly provided by Dr. Alan Davidson (Pawluk et al., 2016; Supplementary Table S1). Single colonies of the strains obtained were cultured overnight, diluted 1:500 into LB and grown to an OD_600_ of 0.3. Subsequently, 0.1 mM IPTG and 0.2% arabinose (Sigma-Aldrich) were added for induction. At an OD_600_ of 0.6, electrocompetent cells were prepared as described previously (Thomason et al., 2014). 50 μl aliquots of each strain were mixed with 1 μl of pETDuet-1 (5 ng/μl) and transferred to a pre-chilled 0.1 cm cuvette prior to electroporation at 1.8 kV (Micropulser, Biorad). Cells were then mixed with 550 μl of warm LB medium and transferred to a culture tube. After 1 h of recovery at 37°C, serial dilutions were plated on LB plates with ampicillin (50 μg/ml), kanamycin (25 μg/ml) and spectinomycin (25 μg/ml). After overnight incubation at 37°C, plates with well-separated colonies were selected and the number of colonies per plate was determined. The Efficiency of Transformation (EOT) was expressed as the ratio between the colony count of the strains carrying wild-type Cas3 and the corresponding strains carrying the Cas3 HD mutation. Assays were performed in triplicate and error bars were calculated as standard error of the mean (SEM).

### Protein Purification

The Type I-Fv complex from *S. putrefaciens* CN-32 was purified as previously described (Gleditzsch et al., 2016) by heterologous expression of the Cascade components (Cas5fv, N-His-Cas7fv, Cas6f, and crRNA) in *E. coli* BL21(DE3). Briefly, cells were grown at 37°C to an OD_600_ of 0.6 before induction with 0.1 mM isopropyl-β-D-1-thiogalactopyranoside (IPTG). Cells were then grown overnight at 18°C and collected by centrifugation at 10.000 × g for 30 min at 4°C. After resuspension in Lysis Buffer (Supplementary Table S2), the cells were disrupted by three rounds of homogenization in a Microfluidizer LM10 (Microfluidics). Lysates were cleared by centrifugation at 38,700 × g at 4°C, and the supernatant was loaded onto a 5 ml HisTrap HP column (GE Healthcare) for Ni-NTA affinity chromatography. After a wash of 5 column volumes (CV) with Wash Buffer, bound protein was eluted with a gradient of 5 CV of Elution Buffer (Supplementary Table S2). Protein-containing fractions were concentrated to a volume of 2 ml, loaded onto a HiLoad 16/600 Superdex 200 pg column (GE Healthcare) and eluted with Size Exclusion buffer (Supplementary Table S2). In this way, Cascade complexes were separated from incomplete complexes, such as Cas7fv filaments and Cas7fv-Cas5fv dimers (Supplementary Figure S2).

The Type I-F complex of *S. baltica* OS195 carrying a His-tagged variant of Cas7f was obtained following the same protocol as for the I-Fv Cascade. I-F and I-Fv Cascade formation was corroborated by SDS-PAGE, Urea-PAGE and mass spectrometry (Supplementary Figure S2). The Cascade solutions were concentrated to 10 μM before use.

His-tagged AcrF7 was overproduced in BL21(DE3) cells and purified following the protocol for Cascade, with adjusted buffers (AcrF7 buffers, Supplementary Table S2) and the omission of the size exclusion chromatography step. Instead, after Ni-NTA affinity chromatography, protein-containing fractions were dialyzed into SEC buffer overnight at 4°C and diluted to 20 μM working stocks. The purity of the AcrF7 preparations was determined by SDS-PAGE (Supplementary Figure S2).

### Oligonucleotide Design

To follow the binding of the effector complex to its target, one of the interactors (effector complex or target DNA) needs to be immobilized to the biosensor as a bait. Choosing the smaller molecule is recommended, as the immobilization of a large protein complex could result in steric clashes that impair proper binding. In addition, if using the bigger molecule as bait, the wavelength shift elicited by the binding of the smaller molecule could be difficult to measure, since the thickness of the bio-layer would not change sufficiently. Therefore, in this work we used the target oligonucleotide as a bait.

A streptavidin-coated bio-layer was chosen to measure the interaction, as biotin-labeled oligonucleotides are commercially available. In order to give the oligonucleotide flexibility, we added a triethylenglycol (TEG) spacer between the biotin residue and the first nucleotide. The Biotin-TEG tag was placed at the 5′-end of the oligonucleotide.

The target oligonucleotide was designed considering the requirements of the CRISPR-Cas complex under study. For Type I-Fv and I-F, the effector complexes bind dsDNA complementary to a 32 nt spacer located on the crRNA, and recognize a GG PAM on the 3′-end of the target strand (Wiedenheft et al., 2011; Dwarakanath et al., 2015). As a dsDNA target is needed, the oligonucleotides containing the target sequence and the PAM were designed as long single-stranded molecules with complementary arms. To promote the formation of the intramolecular duplex, a central flexible 5 nucleotide long thymidine loop was added (Figure 1). The arms were predicted to anneal at room temperature by the RNAstructure web tool (Reuter and Mathews, 2010). All variations used in this work were generated following these principles.

Lyophilized oligonucleotides (Sigma-Aldrich) were dissolved in water to a concentration of 100 μM and stored at −20°C. Working dilutions were made immediately before use. For each experiment, oligonucleotides were further diluted to 50 μM with 2X SEC buffer to match the protein samples (Supplementary Table S2). Oligonucleotide sequences are described in Supplementary Table S3.

### Bio-Layer Interferometry

Measurements were performed on the BLItz platform (FortéBio) using High Precision Streptavidin (SAX) Biosensors (FortéBio). The protocol provided by the BLItz Pro software in the Advanced Kinetics module was modified as indicated on Table 1.

**TABLE 1:** BLI measuring protocol for Cascade-dsDNA interactions.

Step type	Sample type	Position	Duration (s)
Initial baseline	Buffer	Tube	30
Loading	Oligonucleotide	Drop	120
Baseline	Buffer	Tube	30
Association	Protein complex	Drop	300
Dissociation	Buffer	Tube	180

The biosensor was hydrated in a 96-well plate for 10 min. Wells contained 200 μl aliquots of the same SEC buffer that was used for protein purification, as slight changes in buffer composition might lead to false shifts.

As a starting point, non-specific interactions of the protein complex with the biosensor were tested. To this end, no oligonucleotide was added at the loading step. The binding assay was started by placing a hydrated biosensor on the reading tip of the BLItz instrument and incubating it in a 500 μl black assay tube containing 400 μl of SEC buffer. Next, 4 μl of buffer was placed in the drop holder of the machine and the biosensor was shifted into this position. After the loading step, the biosensor was shifted back to the buffer-containing assay tube for baselining. Between samples, the drop holder was always washed once with 4 μl of 0.5 M NaOH and twice with distilled water, and dried with precision wipes (Kimberly Clark). 4 μl of each protein sample (10 μM AcrF7, I-F, or I-Fv Cascades) were loaded independently into the drop holder and the interaction with the bio-layer was recorded until an equilibrium was reached (300 s). Afterward, the dissociation of the complexes was monitored by transferring the biosensor back into buffer. Biosensors were discarded after each measurement.

All proteins tested produced a binding signal in the absence of immobilized DNA, indicating non-specific binding to the biosensor. Therefore, 0.1 μM BSA and 0.01% Triton X-100 were added to the binding buffer as blocking reagents. In this modified buffer, non-specific interactions were reduced to background levels, enabling the specific detection of dsDNA binding.

**FIGURE 2:**
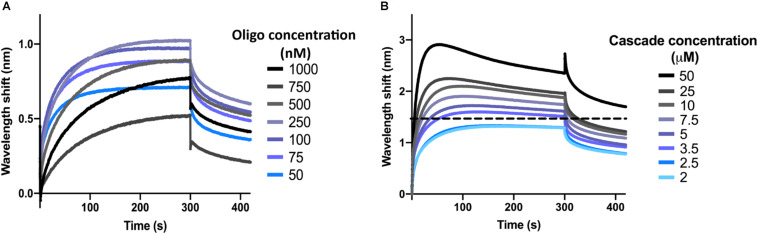
Determination of optimal oligonucleotide concentrations and saturating protein concentrations. **(A)** The wavelength shift (nm) generated by the addition of 1 μM Type I-Fv Cascade to different concentrations of complementary dsDNA oligonucleotide was recorded for 5 min. At an initial concentration of 100 nM of oligonucleotide, the reaction reached equilibrium (plateau) and produced the strongest signal. **(B)** Different concentrations of Type I-Fv Cascade were tested for binding to complementary dsDNA (immobilized at a concentration of 100 nM) for 5 min. The saturating concentration was established as the lowest concentration at which the binding curve reached a stable plateau (blue line). Higher concentrations did not further increase the wavelength shift at equilibrium or caused deviations from the expected binding behavior (as depicted above for 3.5 μM by the dotted line).

Next, the optimal concentration of oligonucleotide was determined. To this end, the dsDNA target oligonucleotide was diluted to concentrations ranging from 1 μM to 50 nM in SEC buffer with 0.1 μM BSA and 0.01% Triton X-100 (BLI buffer). Biosensors were hydrated and the interactions were measured as described above for the control reactions, with SEC buffer being replaced by BLI buffer. At the loading step, 4 μl of oligonucleotide sample were added into the drop holder and loaded onto the biosensor. In each case, the effective concentration of oligonucleotide bound to the biosensor is unknown. For the association step, 1 μM of I-Fv Cascade was used to follow the interaction. The experiment was repeated for each concentration of oligonucleotide, maintaining a constant Cascade concentration. When incubating the biosensor with 100 nM of oligonucleotide, Cascade binding produced the highest wavelength shift at equilibrium (Figure 2A). This concentration was therefore selected for further experiments. Lower concentrations led to lower wavelength shifts due to an unsaturated bio-layer, while higher concentrations showed the same behavior due to an oversaturation, which can result in steric hindrance of the binding of complexes.

Finally, for each Cascade complex, the saturating protein concentration was established. This step is important both for qualitative experiments that need to be performed at non-saturating concentrations, as well as for quantitative experiments, in which saturation needs to be reached to determine the kinetics of the binding reaction.

After immobilization of oligonucleotide (initial concentration of 100 nM), the binding of Cascade was analyzed at concentrations between 50 and 2 μM (Figure 2B). Concentrations above 2.5 μM led to non-ideal binding behavior, as reflected by a decrease in the wavelength shift after initial maximal binding at the association step (instead of a stable plateau), indicating protein aggregation. Therefore, concentrations below 2.5 μM were used for further experiments. Other deviations and a troubleshooting guide can be found elsewhere (Sultana and Lee, 2015).

All binding experiments were performed in duplicates. The concentration of the Cascade complexes and anti-CRISPR protein were determined independently for each experiment by the Bradford Protein Assay, and both the proteins and oligonucleotides were freshly diluted in BLI buffer before each replicate. A loss of the signal between replicates or the measurement of non-ideal binding transients, such as in Figure 2B, were taken as indications of complex degradation. In this cases, new protein and oligonucleotide samples were prepared and re-analyzed.

## Results and Discussion

### Determination of dsDNA-Effector Complex Binding Kinetics

**FIGURE 3:**
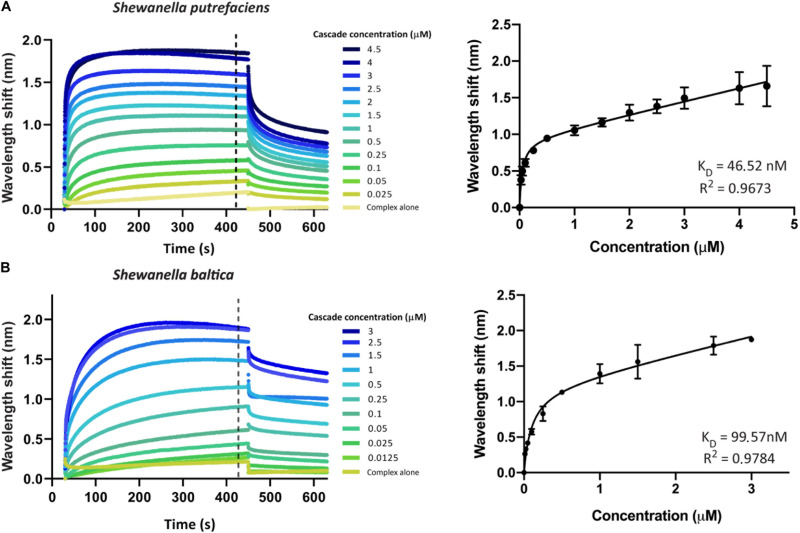
Determination of the equilibrium dissociation constants (K_D_) of the interactions of Type I-Fv and I-F CRISPR-Cas complexes with target dsDNA. Wavelength shift (nm) generated by the addition of Type I-Fv Cascade from *S. putrefaciens* CN-32 **(A)** or Type I-F from *S. baltica* OS195 **(B)** to complementary dsDNA oligonucleotide immobilized on a streptavidin biosensor at a concentration of 100 nM. Binding was followed for 7 min in order to reach equilibrium. Afterward, the protein-bound biosensor was incubated for 3 min in buffer to measure the dissociation reaction. The interaction of the complexes with the dsDNA-free biosensor is shown as control (Complex alone). The wavelength shifts recorded at 420 s after the start of binding were plotted against the corresponding complex concentration in order to calculate the respective K_D_ values. Data were fitted to the non-linear equation: Y = B_max_*X/(K_D_ + X) + NS*X, where B_*max*_ is the maximum wavelength shift and NS the slope of the non-linear component, representing non-specific binding. The coefficients of determination (*R*^2^) and K_D_ values obtained are shown in the graphs for each complex. Binding assays were performed in duplicate. Error bars indicate the Standard Error of the Mean (SEM).

We first aimed to investigate the interaction of Type I-Fv or Type I-F Cascade with an oligonucleotide carrying a sequence with full complementarity to the crRNA (immobilized at a concentration of 100 nM). To this end, we obtained binding transients at a range of different protein concentrations, from saturation to the detection limit. Subsequently, we determined the maximum wavelength shifts reached after equilibration of the reactions (420 s, black dotted line, Figures 3A,B) and plotted the values against the corresponding protein concentrations. The data were then subjected to least-squares regression to determine the K_D_ value.

For both systems, the titration curves obtained did not reach a plateau and contained two components, an exponential and a linear phase. This suggests the existence of both specific interactions with the target and non-specific interactions with the bio-layer (as seen for the “complex alone” samples in Figure 3). To account for this observation, the data were fitted to the following non-linear equation using GraphPad Prism 8.0:

Y=Bmax*XKD+X+NS*X

where Y is the wavelength shift at equilibrium, B_*max*_ the maximum wavelength shift, X the effector complex concentration, K_D_ the equilibrium dissociation constant and NS is the slope of the linear components (accounting for non-specific binding).

Fitting of the data yielded K_D_ values of 46.52 nM for Type I-Fv Cascade (*R*^2^ = 0.97) and of 99.57 nM for Type I-F Cascade (*R*^2^ = 0.98). Interestingly, these K_D_ values are higher than the one reported for the Type I-F system of *Pseudomonas aeruginosa*, obtained by EMSA studies (1 nM; Rollins et al., 2015). This difference in target affinity might correlate with the diversification of the Cascade components, as *S. putrefaciens* carries distinct Cas5fv and Cas7fv subunits and *S. baltica* Type I-F complexes diverged from the *P. aeruginosa* systems (Supplementary Figure S1).

### Analysis of Target Requirements for Binding

For Type I systems, the effector complex recognizes two main features on the target: a correct PAM (Gleditzsch et al., 2019) and a sequence complementary to the crRNA carried by the complex (Barrangou et al., 2007). The optimal PAM described for Type I-F Cascades is a GG pair at the 3′-end of the target strand (Chowdhury et al., 2017; Guo et al., 2017). However, other motifs are also recognized by Type I complexes, albeit with lower efficiency, triggering not only interference but also the acquisition of new spacers into the CRISPR array (primed acquisition) (Hayes et al., 2016). Along the same line, mismatches between the crRNA and the target are allowed to a certain extent (Xue et al., 2015), providing some protection against escape mutations of the targeted elements.

In order to analyze the recognition of these two features, we aimed to investigate the binding of Type I-Fv Cascade to dsDNA oligonucleotides with (i) with a correct PAM and a complementary sequence, (ii) with a TT pair as a PAM and a complementary sequence, and (iii) without sequence complementary to the crRNA and no GG pairs. A TT PAM was selected, because it is the motif present at the end of the repeat in the CRISPR array. Therefore, TT recognition would lead to self-targeting.

**FIGURE 4:**
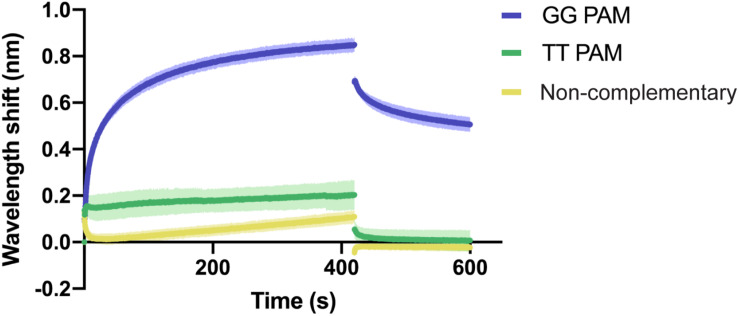
Qualitative analysis of PAM preference by Type I-Fv Cascade. Wavelength shift (nm) generated by the addition of 1.5 μM of Type I-Fv Cascade from *S. putrefaciens* CN-32 to dsDNA oligonucleotides containing a spacer matching to the crRNA and either a GG or a TT PAM. The GG PAM is recognized by the complex, while the TT PAM is not recognized, giving shifts similar to that procured by a non-complementary target. Assays were performed in duplicate. The lighter outlines represent the SEM.

BLI was carried out as described above, using a constant concentration of Type I-Fv Cascade (1.5 μM). As expected, the complex interacts with the complementary target carrying the GG PAM (Figure 4). In contrast, the exchange of the PAM motif to TT strongly impairs the interaction and only allows for transient binding of the Cascade complex, as indicated by its rapid dissociation during the washing step (425 s). A similar binding behavior and even lower affinity was observed for the non-complementary oligonucleotide. The lower shift could be attributed to the absence of PAMs in the oligonucleotide.

As a second qualitative test, we set out to determine the effect of dsDNA mismatches (creating DNA “bubbles”) on the binding of the Type I-Fv Cascade. After the PAM is recognized during interference, the effector complex proceeds to unwind the dsDNA to enable crRNA-DNA pairing, leading to the formation of an R-loop structure. The opening of the target has a directionality, starting from the PAM-proximal site (Huo et al., 2014; Rutkauskas et al., 2015; Xiao et al., 2017). This opening requires the large subunit, but it has been reported that Type I-E complexes without the large subunit can still bind dsDNA targets with mismatched sequences (Huo et al., 2014).

**FIGURE 5:**
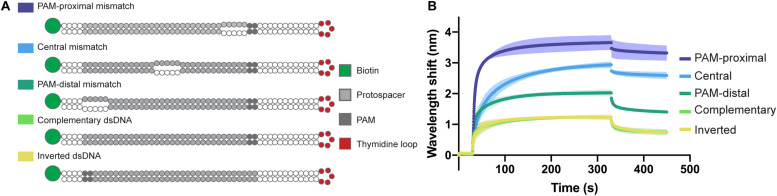
Influence of dsDNA mismatches on Type I-Fv Cascade binding. **(A)** Schematic structures of dsDNA oligonucleotides used in the binding assay. Targets are either fully hybridized or contain 5 nt mismatches at positions in the protospacer close to the PAM, in the centre or at the PAM-distal end. **(B)** Wavelength shift (nm) generated by the addition of 1.5 μM Type I-Fv Cascade from *S. putrefaciens* CN-32 to the indicated dsDNA oligonucleotides. Assays were performed in duplicate. The lighter outlines represent the SEM.

In order to confirm the directionality of the opening process and to test the affinity of the Type I-Fv complex for different bubbled substrates, we designed variants of the complementary dsDNA oligonucleotide. The alternative targets carry 5 nt mismatches either right next to the PAM, in the center of the protospacer, or at the PAM-distal end of it. In addition, as the dsDNA oligonucleotide has a 5 nt thymidine loop, we also inverted the protospacer to test whether the proximity of the mismatch to the loop region would affect the binding behavior (Figure 5A).

The Type I-Fv Cascade showed higher affinity for the target with the PAM-proximal bubble, with increasingly lower signals when binding targets with central and distal bubbles. Nevertheless, all constructs produced a higher wavelength shift than the fully annealed substrate, indicating that mismatches along the target facilitate binding. This observation can be explained by the reduced energy required to open the dsDNA substrates up. Furthermore, these results also corroborate the directionality of the binding process for this minimal system. The target with an inverted protospacer produced a shift similar to the one of the regular protospacer, ruling out an effect of the position of the thymidine loop (Figure 5B).

This system opens the possibility to also study other variables by simply modifying the oligonucleotides, such as the effect of mismatches between crRNA and dsDNA, thus facilitating the identification of seed sequences. Furthermore, it would be possible to obtain further insights into the mechanism of PAM recognition by designing targets with modified nucleosides. For example, the exchange of the GG pair by inosines or other base-pairing alternatives could help elucidate whether there is a strand bias for PAM recognition, as reported for the Type I-F Cascade (Rollins et al., 2015).

### Evaluation of the Effect of Anti-CRISPR Proteins on the Binding of Effector Complexes

BLI offers the possibility to follow the interaction of Cascade complexes with their targets in real time. This property can be exploited to study how additional players influence the binding events. Exemplarily, we show the effect of an Anti-CRISPR protein, AcrF7 from *Pseudomonas aeruginosa*, on the binding of the Type I-Fv and Type I-F Cascades.

**FIGURE 6:**
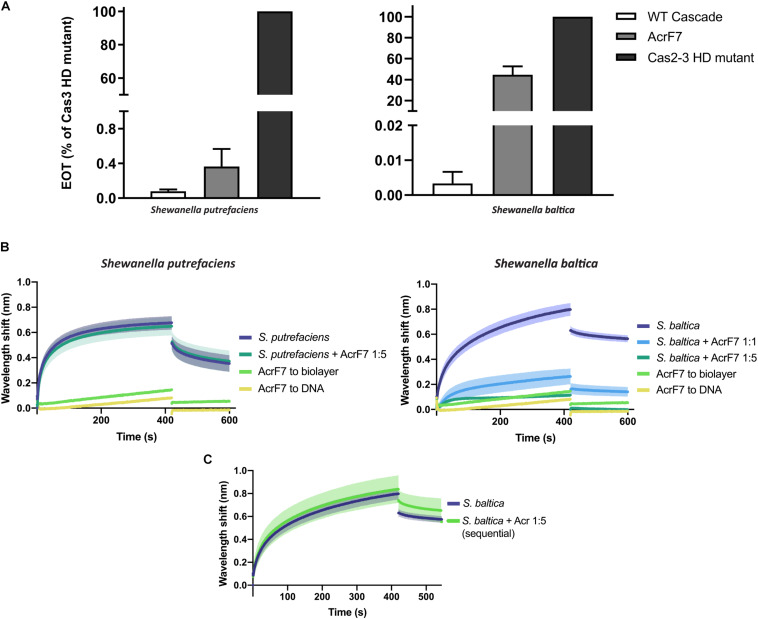
Effect of Anti-CRISPR (Acr) proteins on the binding of CRISPR-Cas complexes to dsDNA. **(A)** Efficiency of Transformation Assays (EOTs) of CRISPR-Cas systems I-Fv from *S. putrefaciens* CN-32 (left) and I-F from *S. baltica* OS195 (right) expressed in *E. coli* BL21-AI. The activity of the effector complexes was tested when expressed alone (WT Cascade) or co-expressed with AcrF7 from *Pseudomonas aeruginosa* (Pawluk et al., 2016). EOT equals to the colony ratio between the strain of interest and its corresponding Cas2/3 HD mutant strain, presented as percentages. Error bars represent the SEM. Three replicates were quantified. **(B)** Wavelength shift (nm) generated by the binding of 500 nM of either Type I-Fv (left) or Type I-F (right) Cascades to complementary oligonucleotide. In order to test the effect of AcrF7, the protein was mixed with Cascades at a 1:1 molar ratio or at a 5-fold molear excess (1:5). Samples were incubated at room temperature for 10 min before analysis. The wavelength shift elicited by the binding of AcrF7 to the dsDNA in the absence of Casade complexees is shown as a control (AcrF7 to DNA). Assays were performed in duplicate. The lighter outlines represent the SEM. **(C)** Wavelength shift (nm) induced by the binding of 500 nM of *S. baltica* I-F Cascade to dsDNA oligonucleotide. The effect of AcrF7 on complexes already bound to dsDNA was tested by incubation of the biosensor in BLI buffer containing 2.5 μM AcrF7 (400 s). Assays were performed in duplicate. The lighter outline represents the SEM.

AcrF7 has been classified as a broad-range Type I-F Acr, as it is able to block the I-F system of both *P. aeruginosa* and *Pseudomonas atrosepticum* (Pawluk et al., 2016). So far, the mechanism behind this inhibition is unknown. Here, we show that it is also able to affect plasmid targeting by the *S. baltica* I-F system when co-expressed in *E. coli*. By contrast, this Acr is not able to significantly obstruct the Type I-Fv complex *in vivo* (Figure 6A).

In order to obtain further insights into this interaction, we purified a His-tagged version of AcrF7 and tested its effect in BLI assays. As a control, we first tested for an interaction of the Acr (5 μM) with unmodified biosensors, which did not produce any significant non-specific binding signal. As a second control, we analyzed the binding of AcrF7 (500 nM) to the dsDNA complementary oligonucleotide. The wavelength shift obtained in this case was very low and rapidly returned to baseline during the dissociation step, indicative of a weak non-specific interaction (Figure 6B).

The BLItz platform has a detection limit of 10 kDa, while the other BLI platforms, such as Octet (FortéBio), can measure wavelengths shifts generated by molecules as small as 150 Da (Sultana and Lee, 2015). AcrF7 has a molecular weight of 10 kDa, including the His-tag. Therefore, when using the BLItz system to study the inhibition, most of the binding signal will depend on the effector complex. For this reason, we first measured the effect of AcrF7 by pre-mixing it with Cascade at different molar ratios and comparing the result with the binding of the complex alone.

In agreement with the *in vivo* assays, the target binding affinity of Type I-Fv Cascade is not affected by the presence of AcrF7 (Figure 6B, left column), as the wavelength shift observed for the complex alone (500 nM) does not change after pre-incubation with a 5-fold excess AcrF7. By contrast, the binding affinity of the Type I-F complex is altered by the presence of the inhibitor (Figure 6B, right column), with an ∼5-fold reduction of the equilibrium binding level at a 1:1 molar ratio, suggesting that AcrF7 impairs dsDNA recognition by the complex. Furthermore, when the complex is exposed to an excess of AcrF7 (1:5 ratio), the interaction is virtually abolished, with the signal reaching the background levels obtained for by the interaction between AcrF7 and the biosensor.

In the next step we took advantage of the modularity of the BLI protocol and explored the ability of AcrF7 to interact with Cascades that were already bound to a target. To this end, we modified the dissociation step of the protocol. After the binding of I-F Cascades to dsDNA targets, the biosensor was incubated in buffer containing AcrF7 (2.5 μM) instead of BLI buffer alone, as used for the control (Figure 6C). In this case, no reduction in the binding activity was observed, suggesting that AcrF7 must act before Cascade complexes find a target. The inhibitory effect of AcrF7 is similar to that of the Type I-F anti-CRISPR proteins AcrF1 and AcrF2. AcrF1 is a small protein that binds the *P. aeruginosa* I-F Cascade specifically between Cas7f subunits, while AcrF2 binds the interface of the 5′-proximal Cas7fv subunit and Cas8f. The interaction of Cascade with these inhibitors consequently prevents both PAM recognition and crRNA-DNA interactions (Bondy-Denomy et al., 2015; Chowdhury et al., 2017; Peng et al., 2017).

Taken together, our results corroborate the resistance of the Type I-Fv complex to AcrF7 and provide insights into the interplay between the inhibitor and the Type I-F complex. They show that AcrF7 can be classified as an inhibitor of the Cascade DNA-binding activity, working on a broader range of Type I-F systems than previously reported. Furthermore, this example highlights the usefulness of BLI to characterize anti-CRISPR proteins, and it shows that BLI can be used to quickly identify the mechanisms of action of other natural or synthetic CRISPR-Cas inhibitors.

Similarly, this method is suitable to characterize the interactions of Cascade complexes with other proteins. For instance, it may be used to further analyze Type I CRISPR-Cas systems, including the recruitment and cleavage activity of the Cas3 nuclease or the interplay with the Cas1-Cas2 adaptation complex, since both of these processes require the previous binding of Cascade to a target.

## Summary and Outlook

As future studies uncover more CRISPR-Cas effector complexes and potential inhibitors, it will be crucial to study the targeting and inhibition potential of these proteins in a fast and reliable manner. BLI has the advantage of measuring interactions with only one labeled player and providing real-time results. Here, we showed how BLI can be used to answer qualitative questions, such as PAM identification, but also provide information on the kinetics of the interactions and the underlying mechanisms. The kinetic values obtained can be corroborated with complementary techniques, such as MicroScale Thermophoresis (MST), as each platform has different levels of accuracy depending on the experimental design. The use of several techniques is, therefore, highly recommended when precise values are needed. In conclusion, BLI offers a practical alternative for studying the interaction between CRISPR-Cas complexes and nucleic acids.

## Data Availability Statement

All datasets generated for this study are included in the article/Supplementary Material.

## Author Contributions

HM-E and LR designed the experiments. HM-E and MO-V performed the experiments. HM-E, MO-V, MT, and LR analyzed the data. NS cloned the *S. baltica* Type I-F CRISPR-Cas system and established the purification of the recombinant effector complex. HM-E wrote the manuscript with input from LR. All authors reviewed and edited the manuscript.

## Conflict of Interest

The authors declare that the research was conducted in the absence of any commercial or financial relationships that could be construed as a potential conflict of interest.
