# Supplementary material for: Bio-Layer Interferometry Analysis of the Target Binding Activity of CRISPR-Cas Effector Complexes
Source: Front Mol Biosci. 2020 May 27;7:98. doi: 10.3389/fmolb.2020.00098 (PMC7266957; doi:10.3389/fmolb.2020.00098)
Supplement: Supplementary file 1 [file Table_1.DOCX]

**Supplementary Table I.** Plasmids used in this study. Plasmids were used for protein purification (white) and EOT assays (grey).

| **Plasmid Name** | **Description** | **Source/**  **Reference** |
| --- | --- | --- |
| pCascadeSP | pRSFDuet-1 containing the genes for His-Cas7fv, Cas5fv and Cas6f from *S. putrefaciens* CN-32. Kan^R^. | Gleditzsch et al., 2016 |
| pCRISPRsp4 | pUC19 containg a T7 polymerase promoter and the repeat-spacer4-repeat sequence from *S. putrefaciens* CN-32. The 8nt handle for Cas5fv was deleted from the second repeat. Amp^R^. | Gleditzsch et al., 2016 |
| pCascadeSB | pRSFDuet-1 containing the genes for Cas8f, Cas5f, His-Cas7f and Cas6f from *S. baltica* OS195. Kan^R^. | This work |
| pAcrF7 | pRSFDuet-1 containing the gene for His-AcrF7 (ACD38920.1). Kan^R^. | This work |
| pCasSP | pRha containing the genes for Cas2-3, Cas7fv, Cas5fv and Cas6f from *S. putrefaciens* CN-32. Cam^R^. | This work |
| pCasHDSP | pRha containing the genes for Cas2-3 HD mutant, Cas7fv, Cas5fv and Cas6f from *S. putrefaciens* CN-32. Cam^R^. | This work |
| pCRISPRamp | pCDFDuet-1 containing the repeat-spacer anti-amp-repeat sequence. Repeats are from the CRISPR array of *S. putrefaciens* CN-32. Spec^R^. | Pausch et al., 2017 |
| pCascadeSBCam | pACYCDuet-1 containing the genes for Cas8f, Cas5f, Cas7f and Cas6f from *S. baltica* OS195. Cam^R^. | This work |
| pCas3SB | pCRISPRamp modified by the addition of the gene coding for Cas2-3 from *S. baltica* OS195. Spec^R^. | This work |
| pCas3HDSB | pCRISPRamp modified by the addition of the gene coding for Cas2-3 HD mutant from S. *baltica* OS195. Spec^R^. | This work |
| pETDuet-1 | Target Plasmid. Amp^R^. | Novagen |
