# Supplementary material for: Bio-Layer Interferometry Analysis of the Target Binding Activity of CRISPR-Cas Effector Complexes
Source: Front Mol Biosci. 2020 May 27;7:98. doi: 10.3389/fmolb.2020.00098 (PMC7266957; doi:10.3389/fmolb.2020.00098)
Supplement: Supplementary file 2 [file Table_2.DOCX]

**Supplementary Table II.** Buffers for protein purification.

| **Protein Sample** | **Buffer Name** | **Components** |
| --- | --- | --- |
| *S. putrefaciens S. baltica* | Lysis Buffer | 50 mM Tris-HCl (pH 7.5) |
| Cascade |  | 300 mM NaCl |
|  |  | 10 mM MgCl_2_ |
|  |  | 1mM Dithiothreitol (DTT) |
|  |  | 10% Glycerol |
|  | Wash Buffer | Lysis Buffer + 20 mM imidazole |
|  | Elution Buffer | Lysis Buffer + 500 mM imidazole |
| AcrF7 | Lysis Buffer | 20 mM Tris-HCl (pH 7.5) |
|  |  | 250 mM NaCl |
|  |  | 1 mM DTT |
|  | Wash Buffer | Lysis Buffer + 20 mM imidazole |
|  | Elution Buffer | Lysis Buffer + 500 mM imidazole |
| All samples | SEC Buffer | 50 mM HEPES-KOH (pH 7.3) |
|  |  | 150 mM NaCl |
|  |  | 1 mM DTT |
