# Supplementary material for: Bio-Layer Interferometry Analysis of the Target Binding Activity of CRISPR-Cas Effector Complexes
Source: Front Mol Biosci. 2020 May 27;7:98. doi: 10.3389/fmolb.2020.00098 (PMC7266957; doi:10.3389/fmolb.2020.00098)
Supplement: Supplementary file 3 [file Table_3.DOCX]

**Supplementary Table III.**  Biotinylated oligonucleotides used on this study. The thymidine loop is shown in blue.

| **Oligonucleotide Name** | **Sequence (with 5’ Biotin-TEG tag)** |
| --- | --- |
| Complementary | CGCCGGTTATAGGTTTGCGCGTCTTGCTGGGCGATAGGACGTGGGATATCTTTTTGATATCCCACGTCCTATCGCCCAGCAAGACGCGCAAACCTATAACCGGCG |
| Non-complementary | GCTGAACATGATACGCTTCAATAGTTAGATGACGCAGTAGTTATGATATCTTTTTGATATCATAACTACTGCGTCATCTAACTATTGAAGCGTATCATGTTCAGC |
| TT PAM | CGCCGGTTATAGGTTTGCGCGTCTTGCTGGGCGATATTACGTGGGATATCTTTTTGATATCCCACGTAATATCGCCCAGCAAGACGCGCAAACCTATAACCGGCG |
| PAM-proximal mismatch | CGCCGGTTATAGGTTTGCGCGTCTTGCTGGGCGATAGGACGTGGGATATCTTTTTGATATCCCACGTCCGCGTACCCAGCAAGACGCGCAAACCTATAACCGGCG |
| Central mismatch | CGCCGGTTATAGGTTTGCGCGTCTTGCTGGGCGATAGGACGTGGGATATCTTTTTGATATCCCACGTCCTATCGCCCAGCAAGATAAAAAAACCTATAACCGGCG |
| PAM-distal mismatch | CGCCGGTTATAGGTTTGCGCGTCTTGCTGGGCGATAGGACGTGGGATATCTTTTTGATATCCCACGTCCTATCGCCCAGCAAGACGCGCAAACCTAGGCTTGGCG |
| Inverted protospacer | CGCCCCTATCGCCCAGCAAGACGCGCAAACCTATAACCACGTGGGATATCTTTTTGATATCCCACGTGGTTATAGGTTTGCGCGTCTTGCTGGGCGATAGGGGCG |
