# Supplementary material for: Bio-Layer Interferometry Analysis of the Target Binding Activity of CRISPR-Cas Effector Complexes
Source: Front Mol Biosci. 2020 May 27;7:98. doi: 10.3389/fmolb.2020.00098 (PMC7266957; doi:10.3389/fmolb.2020.00098)
Supplement: Supplementary file 4 [file Data_Sheet_1.pdf]

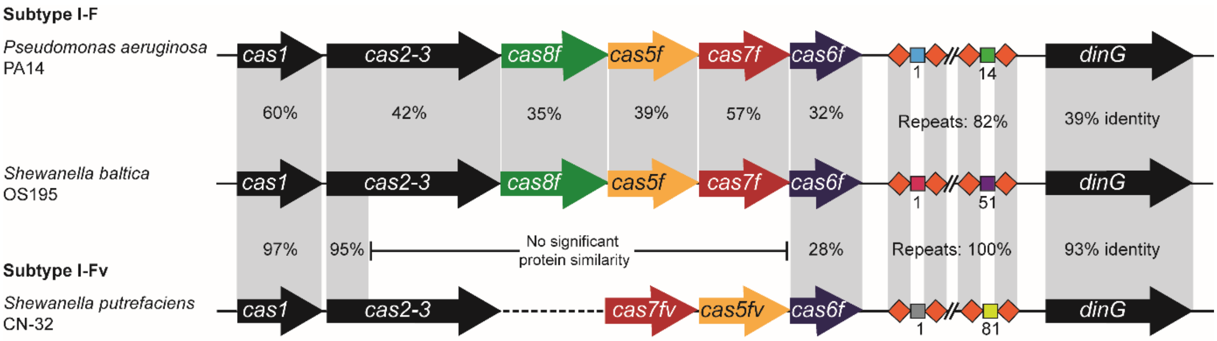

Supplementary Figure 1. Comparison of the CRISPR-Cas systems from *P. aeruginosa* PA14 and *S. putrefaciens* CN-32 against the I-F system from *S. baltica* OS195. Schematic representation of the CRISPR-*cas* loci. The arrows indicate the *cas* and related genes in comparative lengths. Cascade genes are multicolored. The CRISPR arrays are indicated as alternating rhombi (orange, CRISPR repeats) and squares of various colors (spacers). Grey highlights indicate sections of significant protein sequence identity and high sequence complementarity (for the repeats).
