# Supplementary material for: Bio-Layer Interferometry Analysis of the Target Binding Activity of CRISPR-Cas Effector Complexes
Source: Front Mol Biosci. 2020 May 27;7:98. doi: 10.3389/fmolb.2020.00098 (PMC7266957; doi:10.3389/fmolb.2020.00098)
Supplement: Supplementary file 5 [file Data_Sheet_2.PDF]

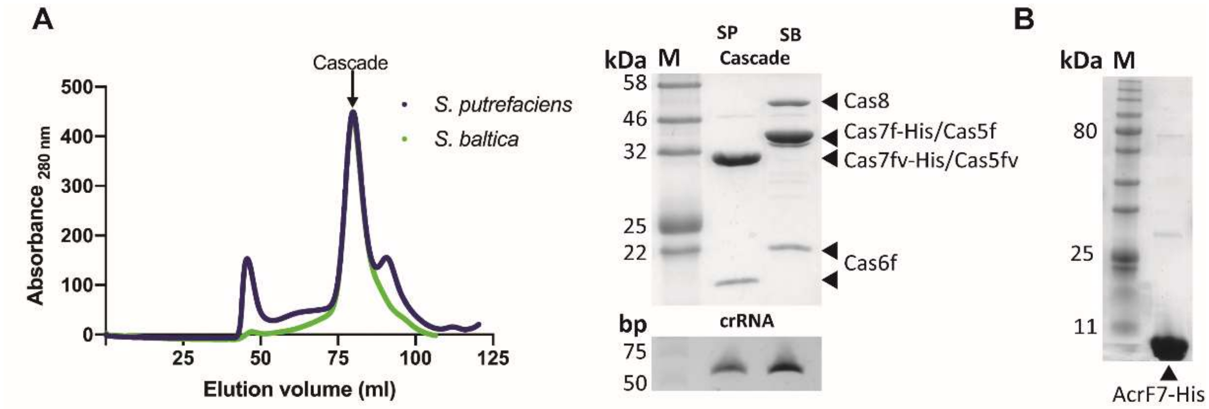

Supplementary Figure 2. Purification of Type I-F/I-Fv Cascades and AcrF7. (A) Chromatogram obtained after IMAC and SEC purification of Type I-F Cascade from *S. baltica* OS195 and Type I-Fv Cascade from *S. putrefaciens* CN-32, heterologously expressed in *E. coli* BL21-AI. For both complexes, the Cas7 protein carried an N-terminal hexahistidine (His-) tag. SEC was performed using a HiLoad Superdex 16/600 200 pg column (GE Healthcare). The composition of the peak samples (asterisks) was analyzed by SDS-PAGE and Urea-PAGE, which confirmed the presence of all Cascade components and crRNA. SB = *S. baltica*, SP= *S. putrefaciens*. (B) SDS-PAGE of the AcrF7 sample obtained after IMAC and overnight dialysis.
